# Quantifying Viral Lysis in Microalgae Using Cell‐Free rRNA

**DOI:** 10.1002/mbo3.70402

**Published:** 2026-09-09

**Authors:** Saki Kikuya, Yuji Tomaru, Keizo Nagasaki, Daichi Morimoto, Yuki Yamagishi, Hiroyuki Ogata, Hisashi Endo

**Affiliations:** ^1^ Bioinformatics Center Institute for Chemical Research Kyoto University, Gokasho Uji Kyoto Japan; ^2^ Fisheries Technology Institute, Japan Fisheries Research and Education Agency Hatsukaichi Hiroshima Japan; ^3^ Faculty of Science and Technology Kochi University, Monobe‐Otsu Nankoku Kochi Japan

**Keywords:** cell‐free rRNA, lysis rate, marine ecosystems, phytoplankton, viruses

## Abstract

Viral lysis is a major driver of phytoplankton mortality and a key source of dissolved organic matter in the ocean, yet quantitative and taxon‐resolved measurements of this process remain limited for eukaryotic plankton. Here, we demonstrate that cell‐free ribosomal RNA (rRNA) dissolved in the extracellular medium serves as a quantitative proxy for viral lysis in microeukaryotes by combining laboratory infection experiments with a rate‐based modeling framework. We investigated two host–virus systems: the diatom *Chaetoceros tenuissimus* infected by a single‐stranded RNA virus (CtenRNAV) and the raphidophyte *Heterosigma akashiwo* infected by a large double‐stranded DNA virus (HaV). By explicitly accounting for cell‐free rRNA degradation, we quantified a proxy for cell lysis rate from cell‐free 18S rRNA dynamics and observed up to 46‐ and 302‐fold increases in cell‐normalized lysis rates under viral infection. In *C. tenuissimus*, peak lysis preceded the decline in cell abundance, demonstrating that substantial viral lysis occurs while populations are still proliferating. In contrast, *H. akashiwo* exhibited elevated lysis in both early and late infection phases, implying that a fraction of host cells undergoes rapid, nonproductive mortality prior to viral replication, in addition to canonical lytic events. Our study provides the first experimental validation of cell‐free rRNA as a quantitative marker of lytic mortality in microeukaryotes, offering a scalable approach for estimating taxon‐specific mortality in natural plankton communities.

## Graphical abstract

Cell‐free ribosomal RNA (rRNA) dissolved in seawater provides a quantitative proxy for viral lysis in microeukaryotic phytoplankton. Incubation experiments with two distinct host–virus systems revealed marked increases in cell‐free rRNA production per cell, a proxy for lytic mortality, reaching up to 46‐ and 302‐fold relative to noninfected controls. This approach would enable scalable, taxon‐resolved estimation of lytic mortality in natural plankton communities. (The image generated using ChatGPT‐5.5 based on the author's original sketch.)

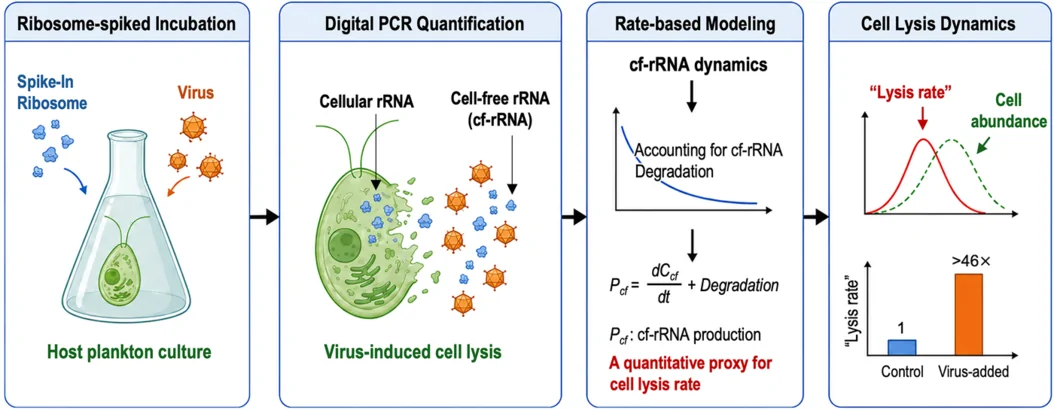

## 1 Introduction

Marine protists account for approximately 30% of oceanic carbon biomass and play central roles in global biogeochemical cycles (Bar‐On and Milo 2019). Among them, phytoplankton act as primary producers, fixing CO_2_ at rates comparable to land plants and sustaining marine food webs (Field et al. 1998). Their populations exhibit rapid turnover, with generation times ranging from hours to days, and their mortality processes strongly influence carbon and nutrient cycling (Fuhrman et al. 2015).

Cell lysis is an important cause of plankton cell death. This process releases cellular contents into the environment as bioavailable dissolved organic matter (DOM), which is rapidly consumed and then partly transformed into recalcitrant DOM through microbial processing (Jiao et al. 2024; Moran et al. 2022). Viruses are responsible for the death of an estimated 10%–40% of marine microbes per day via the so‐called “viral shunt” (Baudoux et al. 2008; Suttle 2007; Vincent et al. 2021). Recent omics studies have suggested that the population dynamics of various plankton lineages may be influenced by viruses (Endo et al. 2020; Xia et al. 2022). Despite the ecological importance of viral lysis, quantifying its contribution to phytoplankton mortality remained difficult because no widely accepted molecular marker indicative of cell lysis had been discovered.

A recent study demonstrated that ribosomal RNA (rRNA) is released in substantial amounts from cells into the extracellular medium via viral lysis of prokaryotes, serving as a molecular marker of virus‐induced lysis (Liu et al. 2025; Liu et al. 2023; Zhong et al. 2023). Since rRNA contains conserved and variable regions, this phenomenon can be applied to estimate taxon‐specific cell lysis in microbial communities by combining amplicon sequencing of both cellular and extracellular fractions. However, this approach (Mortality by Ribosomal Sequencing, MoRS) has not been validated for unicellular eukaryotes. Eukaryotic cells possess complex intracellular organization, including nuclei and organelles, and exhibit diverse viral life strategies, ranging from small RNA viruses to giant DNA viruses. Thus, the concept of MoRS cannot be simply extrapolated to microeukaryotes.

To address this issue, this study examined the feasibility of applying MoRS to microeukaryotes by laboratory infection experiments of two marine algae, the diatom *Chaetoceros tenuissimus* and the raphidophyte *Heterosigma akashiwo*. *C. tenuissimus* is a small phytoplankton (< 10 µm) that can form blooms in coastal waters (Tomaru et al. 2017). Diatom viruses reported to date are classified as single‐stranded RNA (ssRNA) viruses or single‐stranded DNA (ssDNA) viruses. Both are small, spherical viruses with particle sizes around 22–38 nm. They infect specific host diatom species and cause cell lysis. For ssRNA viruses, five species have been reported, including *C. tenuissimus* RNA virus (CtenRNAV), which possesses two open reading frames encoding replication‐associated proteins and capsid proteins (Kimura and Tomaru 2015; Shirai et al. 2008). *H. akashiwo* is a globally distributed mixotrophic plankton species possessing two flagella for mobility. This species is known to form harmful algal blooms in coastal environments. To date, RNA and DNA viruses infecting *H. akashiwo* have been isolated (Nagasaki and Yamaguchi 1997; Tai et al. 2003). This species is infected by several viruses, including *H. akashiwo* virus (HaV), a large double‐stranded DNA (dsDNA) virus that exhibits strain‐specific infectivity and complex host–virus interactions (Funaoka et al. 2023; Nagasaki and Yamaguchi 1998).

**Figure 1:**
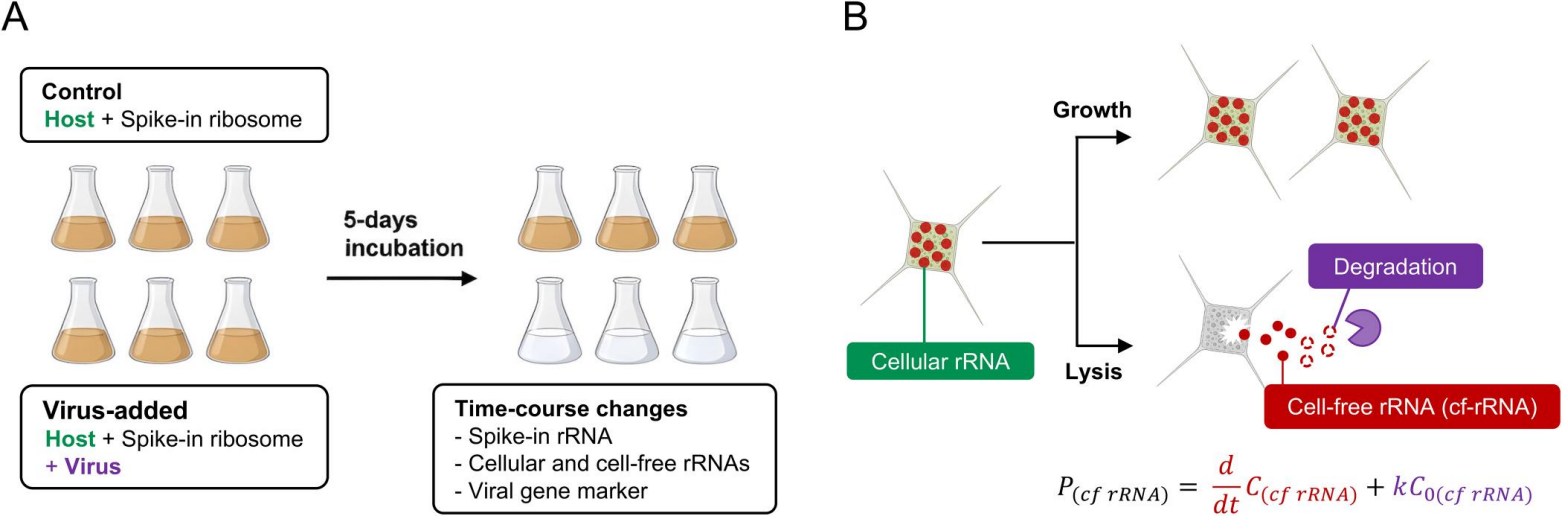
A schematic diagram of this experiment. (A) Design of the incubation system and measured parameters. (B) Concept of a rate equation to quantify cell‐free rRNA production rate (a proxy for cell lysis). rRNA, ribosomal RNA.

Together, these systems represent contrasting host–virus pairs in terms of host physiology and viral genome type, providing an ideal framework for testing the generality of cell‐free rRNA (i.e., rRNA dissolved in the extracellular fraction) as a marker of viral lysis. The primary objective of this study is to experimentally determine whether viral lysis of eukaryotic microalgae leads to the release of rRNA into the dissolved phase. Specifically, we quantify the extent of cell lysis by measuring cell‐free 18S rRNA and develop a rate‐based model that incorporates cell‐free rRNA degradation (Figure 1). This approach establishes a mechanistic basis for using cell‐free rRNA as a molecular indicator of viral lysis in marine eukaryotic phytoplankton.

## 2 Material and Methods

### 2.1 Experimental Conditions and the Host and Virus Strains

Laboratory infection experiments were conducted using two established marine microalgal host–virus systems: the diatom *C. tenuissimus* (strain NIES‐3715) infected by CtenRNAV type‐II (CtenRNAV‐II) (Kimura and Tomaru 2015), and the raphidophyte *H. akashiwo* (strain U13262) infected by HaV strain 120 (HaV120) (Funaoka et al. 2023). These systems were selected to represent contrasting host phylogenetic groups and viral genome types (ssRNA vs. large dsDNA viruses), while allowing direct comparison of viral lysis‐associated cell‐free rRNA dynamics under controlled conditions.

All host cultures were maintained under static conditions at 22°C with a 12‐h light/12‐h dark photoperiod (lights on at 6:00, off at 18:00) and an integrated photon flux density of approximately 200 µE m^−2^ s^−1^. Cultures were grown in sterile artificial seawater–based media and were acclimated for multiple generations prior to infection experiments to ensure stable exponential growth.

For infection assays, host cultures were pre‐cultured in 2 L flasks and harvested during the exponential growth phase. Pre‐cultures were then distributed into six 500 mL polyethylene terephthalate glycol vent‐cap flasks, each containing 200 mL of culture. For each host–virus system, three flasks were assigned to virus‐infected treatments and three to noninfected controls. Viral lysates were added to infected treatments at a multiplicity of infection (MOI) of 10. Control treatments received no virus addition. Flasks were arranged in the incubator to minimize spatial heterogeneity in light exposure and were incubated for 6 days. Samples for downstream analyses were collected daily between 09:00 and 10:00.

The diatom *C. tenuissimus* was cultured in mSWM‐3 medium (Imai et al. 1996). Viral lysates were stored at −80°C prior to use. This experiment was conducted under axenic conditions. The raphidophyte *H. akashiwo* was cultured in IMK medium. Infection assays were initiated at a host cell density above 1 × 10^5^ and 1 × 10^4^ cells mL^−1^ for *C. tenuissimus* and *H. akashiwo*, respectively. Because the HaV120 viral stock was nonaxenic, penicillin–streptomycin solution was added to all treatments at a final concentration of 100 µg mL^−1^ prior to inoculation to suppress bacterial growth.

To quantify viral proliferation, viral titers were determined immediately after viral addition (day 0) and on the final day of incubation (day 5) using the most probable number (MPN) method. At each time point, we performed the MPN assay for a single incubation bottle without biological replication. In addition, to evaluate the cell‐free rRNA degradation constant, purified ribosomes from *Escherichia coli* strain B (New England Biosciences, cat. P0763S) were added to all incubation bottles as spike‐in controls at a final concentration of approximately 2.9 × 10^7^ copies mL^–1^.

### 2.2 Viral Titration Assay

Viral titers were determined using the MPN method (Suttle and Chan 1993). Briefly, 96‐well microplates were inoculated with 150 µL of exponentially growing culture per well. Serial 10‐fold dilutions of viral lysates were prepared, and 100 µL of each dilution was added to eight replicate wells. Plates were incubated at the same temperature and light conditions as described above for 10–14 days. Viral infection was assessed by microscopic examination of host lysis, and viral concentrations were calculated using MPNcalc v1.5.0 based on the number of positive wells at each dilution.

### 2.3 Microscopic Cell Counts and Chlorophyll *a* Measurement

Host cell abundance was determined by manual counting using an inverted light microscope (Olympus CKX53). Prior to sampling, culture flasks were gently swirled to homogenize the suspension. Aliquots were placed on disposable counting chambers (Sekisui Chemical, UR‐157). The specific growth rate $\mu_{i}$ (day^–1^) of the host cells was calculated over each daily interval as

(1) $$\mu_{i}=\frac{\ln\;C_{\mathrm{cell}}\left(t_{i+1}\right)-\ln\;C_{\mathrm{cell}}\left(t_{i}\right)}{∆t},$$

where $C_{\mathrm{cell}}$ represents the host cell concentration, $t_{i}$ and $t_{i+1}$ are the successive sampling times, and ∆t=1 (day). Negative values of $\mu_{i}$ were interpreted as periods during which host losses exceeded host growth (i.e., net decline in cell abundance).

Chlorophyll *a* fluorescence was measured using a Qubit 4 Fluorometer (Invitrogen) with a blue excitation source (470 nm). Chlorophyll *a* concentrations were calculated using a calibration curve generated from standard solutions (CAS RN 479–61–8, Fuji film).

### 2.4 Flow Cytometry

For the *H. akashiwo* experiment, we collected samples (2 mL) for flow cytometry, which were preserved with paraformaldehyde (0.2% in final concentration) and stored in a deep freezer. Bacterial cell abundance in the medium was measured using a CytoFLEX flow cytometer (Beckman Coulter) equipped with a blue (488 nm) laser. The detailed experimental procedure is described elsewhere (Endo et al. 2025).

### 2.5 Cellular and Cell‐Free RNA Extractions and Complementary DNA (cDNA) Synthesis

In this study, RNA retained on a 0.22‐µm filter was operationally defined as the cellular (intracellular) fraction, whereas that passing through the filter was defined as the cell‐free (extracellular) fraction. Culture samples (20 mL) were gently filtered through 47 mm diameter, 0.22 µm pore‐size polycarbonate filters (Isopore GTTP04700, Merck Millipore) under low vacuum pressure (< 0.013 MPa). Filters were immediately immersed in 600 µL RLT buffer containing 1% β‐mercaptoethanol and 0.2 g of 0.1 mm glass beads. Filtrates were collected in sterile 50 mL conical tubes. All samples were stored at –80°C until nucleic acid extraction.

Extractions of cellular and cell‐free RNA were performed following Endo et al. (2025). Briefly, cellular RNA was extracted using the RNeasy Plus Mini Kit (QIAGEN) with minor modifications. To enhance cell lysis, frozen samples were bead‐beaten at 2500 rpm for 50 s, repeated three times with cooling intervals. The buffer RLT was used instead of buffer RLT Plus. Cell‐free RNA was extracted from 20 mL filtrates using the Wizard Enviro Total Nucleic Acid Kit (Promega), following the manufacturer's protocol. This kit employs a column‐based vacuum system optimized for concentrating viral and cell‐free nucleic acids from large volumes of water. RNA concentrations were measured fluorometrically using a Quantus Fluorometer (Promega) with the QuantFluor RNA System. RNA size distributions were assessed using an Agilent TapeStation with either standard RNA or High Sensitivity RNA kits, depending on sample concentration.

Genomic DNA removal and first‐strand cDNA synthesis were performed using SuperScript IV VILO Master Mix with ezDNase Enzyme (Thermo Fisher Scientific), following the manufacturer's instructions.

### 2.6 Digital Polymerase Chain Reaction (PCR) Quantification of rRNA and Viral Marker Genes

Absolute quantification of cDNA from cellular and cell‐free fractions was performed using a QIAcuity Digital PCR system (QIAGEN) with the QIAcuity EG PCR Kit, which enables sensitive and accurate quantification, minimizing the effects of PCR inhibitors and amplification efficiency. Newly designed primer pairs targeting the 18S V4 region were used for *C. tenuissimus* and *H. akashiwo* (Table 1). In addition, 16S rRNA from the *E. coli* strain B was quantified in the cell‐free fractions to assess rRNA degradation in the medium. The viral gene abundances in the cDNA libraries were quantified from cellular and cell‐free fractions using primer pairs specific to the major capsid protein (MCP) gene of CtenRNAV‐II or HaV120. Note that the number of cDNA reflects both cellular transcripts and viral genomes, as CtenRNAV‐II is an RNA virus. The primer sequences and the annealing temperatures were summarized in Table 1. The amplifications were performed with the following conditions: 95°C for 2 min, denaturation at 95°C for 15 s, and annealing/extension for 30 s (at the temperature optimized for each primer pair) for 40 cycles.

**Table 1:** The PCR primer sequences and the annealing temperatures.

Target	Primer name	Sequences (5′–3′)	Product size (bp)	Annealing temperature (°C)
18S rRNA, *Chaetoceros tenuissimus*	18S_E572F	CYGCGGTAATTCCAGCTC	127	59
	18S_Cten652R	CAAGGACGAGGCAAGAGGATTA		
18S rRNA, *Heterosigma akashiwo*	18S_E572F	CYGCGGTAATTCCAGCTC	118	59
	18S_HA689R	GGACGATACAGGTGCAGAC		
16S rRNA, *Escherichia coli* strain B	16S_EC442F	GCGGGGATGAAGGGAGTAAA	195	58
	16S_EC636R	ATGCAGTTCCCAGGTTGAGC		
MCP, CtenRNAV‐II	MCP_CtenRVII_8047F	ATGGACCGATATCCGTGCA	93	59
	MCP_CtenRVII_8131R	AACAGCAGCTGCCGTATTG		
MCP, HaV120	MCP_HaV883F	TCAAGCCATGTACCGAACTG	85	59
	MCP_HaV975R	ATGGACAAACGCTATCGGTC		

Abbreviations: MCP, major capsid protein; PCR, polymerase chain reaction; rRNA, ribosomal RNA.

Concentrations of cDNAs in seawater medium were corrected based on the extraction efficiencies of 21.9% and 83.1% for the cellular and cell‐free fractions, respectively. The extraction efficiencies were adopted from Endo et al. (2025), in which the same extraction protocol was evaluated (Endo et al. 2025).

### 2.7 Quantification of Host Cell Lysis Rates

Lysis rate of host cells was quantified using rate‐based models that describe daily changes in cell‐free rRNA concentrations ($C_{\mathrm{cf}}$) while accounting for rRNA degradation after release into the medium. These models explicitly operate at the daily time scale, corresponding to the sampling time resolution. The degradation rate constant of cell‐free rRNA was characterized daily by temporal changes in spike‐in rRNA concentration ($C_{\mathrm{spike}}$), assuming first‐order kinetics.

(2) $$C_{\mathrm{spike}}\left(t_{i+1}\right)=C_{\mathrm{spike}}\left(t_{i}\right)e^{-k∆t},$$

where k is the degradation rate constant (day^–1^) of cell‐free rRNA.

Temporal change in $C_{\mathrm{cf}}$ reflects the balance between rRNA released from the cells and the degradation in the medium. The temporal dynamics of cell‐free rRNA are described by

(3a) $$\frac{dC_{\mathrm{cf}}}{\mathrm{dt}}=P_{\mathrm{cf}}-{\mathrm{kC}}_{\mathrm{cf}}.$$

By rearranging Equation (3a), the cell‐free rRNA production rate is expressed as

(3b) $$P_{\mathrm{cf}}=\frac{dC_{\mathrm{cf}}}{\mathrm{dt}}+{\mathrm{kC}}_{\mathrm{cf}},$$

where $P_{\mathrm{cf}}$ is the cell‐free rRNA production rate (copies mL^–1^ day^–1^). Since the rRNA concentrations were obtained at daily intervals, we applied a discrete‐time mass balance model over each interval:

(4a) $$C_{\mathrm{cf}}(t_{i+1})=C_{\mathrm{cf}}\left(t_{i}\right)e^{-k∆t}+\frac{P_{\mathrm{cf},i}}{k}(1-e^{-k∆t}),$$

where $P_{\mathrm{cf},i}\;$represents the cell‐free rRNA production rate between $t_{i}$ and $t_{i+1}$. Solving Equation (4a) for $P_{\mathrm{cf},i}$ yields

(4b) $$P_{\mathrm{cf},i}=k\frac{{C_{\mathrm{cf}}\left(t_{i+1}\right)-C}_{\mathrm{cf}}\left(t_{i}\right)e^{-k∆t}}{1-e^{-k∆t}}.$$

This formulation provides a quantitative proxy for cell lysis based on cell‐free rRNA dynamics. In this study, we further define cell‐free rRNA production normalized by host cell abundance as cell‐free rRNA production per cell (cf‐rRNA‐PPC). Under the assumption that cellular rRNA content is relatively constant and that cell lysis is the dominant source of cell‐free rRNA, this metric is proportional to the cell lysis rate.

### 2.8 Statistical Analysis

To evaluate treatment effects on temporal dynamics, we performed a two‐way repeated‐measures analysis of variance (ANOVA) with treatment (control vs. virus‐added) as a between‐subjects factor and time as a within‐subjects factor, treating replicate cultures as subjects. Models included treatment, time, and their interaction, with the interaction term used to assess differences in temporal trajectories. Post hoc comparisons were performed using estimated marginal means, with Holm‐adjusted *p* values. All statistical analyses were conducted using the packages “afex” (https://cran.r‐project.org/web/packages/afex) and “emmeans” (https://cran.r‐project.org/web/packages/emmeans) in R (v4.5.2).

## 3 Results

### 3.1 Host Growth Dynamics With and Without Viruses

**Figure 2:**
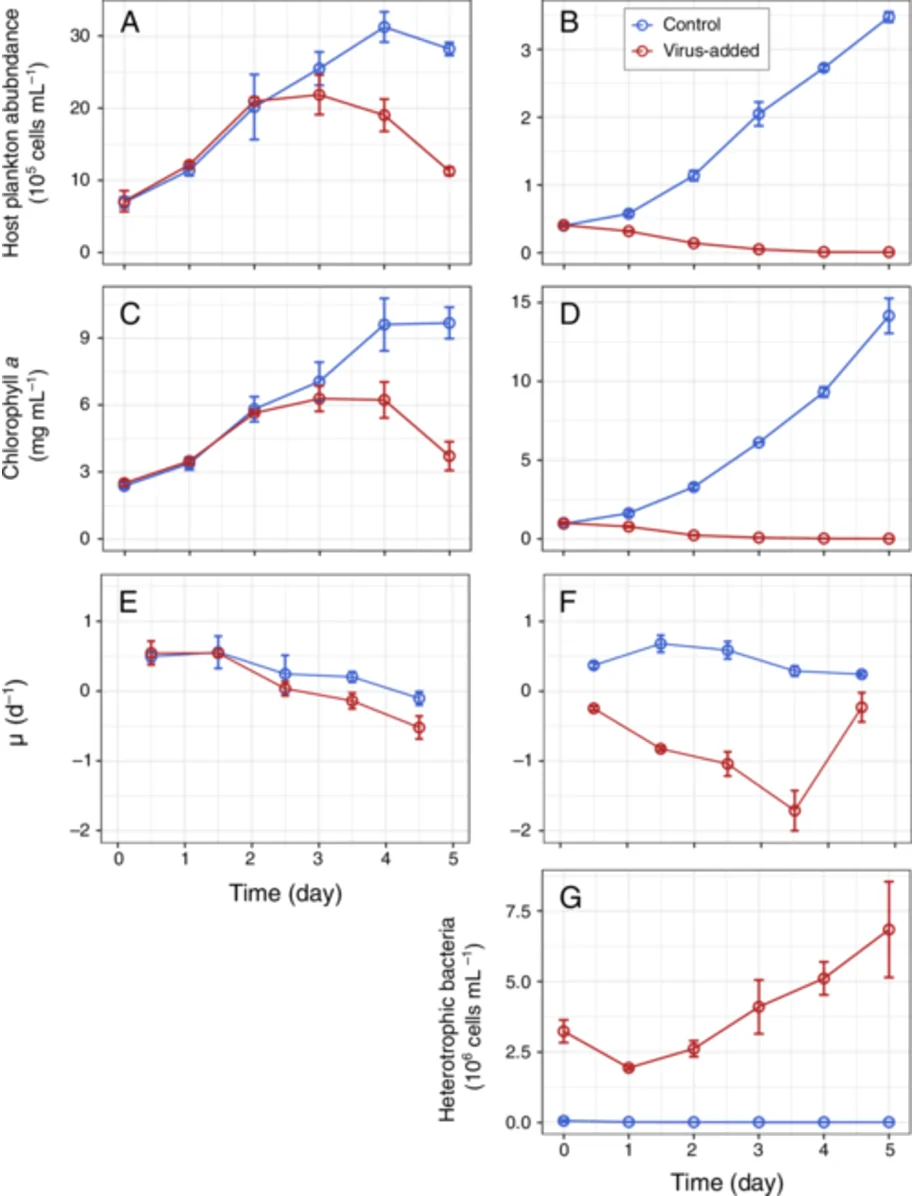
Temporal changes in cell density (A, B), chlorophyll *a* concentration (C, D), specific growth rate (*μ*) of host cells (E, F), and heterotrophic bacterial abundance (G). Left and right panels denote the data from *Chaetoceros tenuissimus* and *Heterosigma akashiwo* experiments, respectively. Heterotrophic bacterial abundance was measured only for the *H. akashiwo* experiment. Error bars denote ±1 SD (*n* = 3).

In *C. tenuissimus*, the initial cell density was 6.97 ± 0.76 × 10^5^ cells mL^–1^ (mean ± standard deviation, SD). In the control treatment, cell density increased until day 4, when it reached a maximum of 3.13 ± 0.21 × 10^6^ cells mL^–1^ (Figure 2A). In the virus‐added treatment, the specific growth rate of the cells was comparable to that of the control until day 3 but the values were significantly lower at later periods (Holm‐adjusted *p* < 0.05) (Figure 2A,E).

In *H. akashiwo*, the initial cell density was 3.97 ± 0.09 × 10^4^ cells mL^–1^ (mean ± SD). In the control treatment, cell densities increased through day 5 and reached a maximum of 3.48 ± 0.08 × 10^5^ cells mL^–1^ (Figure 2B). In the virus‐added treatment, cell density declined immediately after viral addition (Holm‐adjusted *p* < 0.05), and the specific growth rate showed the lowest value between days 3 and 4 (Figure 2B,F).

In both the *C. tenuissimus* and *H. akashiwo* experiments, temporal changes in chlorophyll *a* biomass followed patterns similar to those of cell densities (Figure 2C,D).

We also quantified heterotrophic bacterial abundance in *H. akashiwo* culture bottles. Despite the use of antibiotics, a significantly higher bacterial density (up to 6.84 ± 1.70 × 10^6^ cells mL^–1^, mean ± SD) was detected in the virus‐added treatment (Holm‐adjusted *p* < 0.05), whereas it was negligible in the control (Figure 2G).

### 3.2 Redistribution of rRNA Between Particulate and Dissolved Fractions

**Figure 3:**
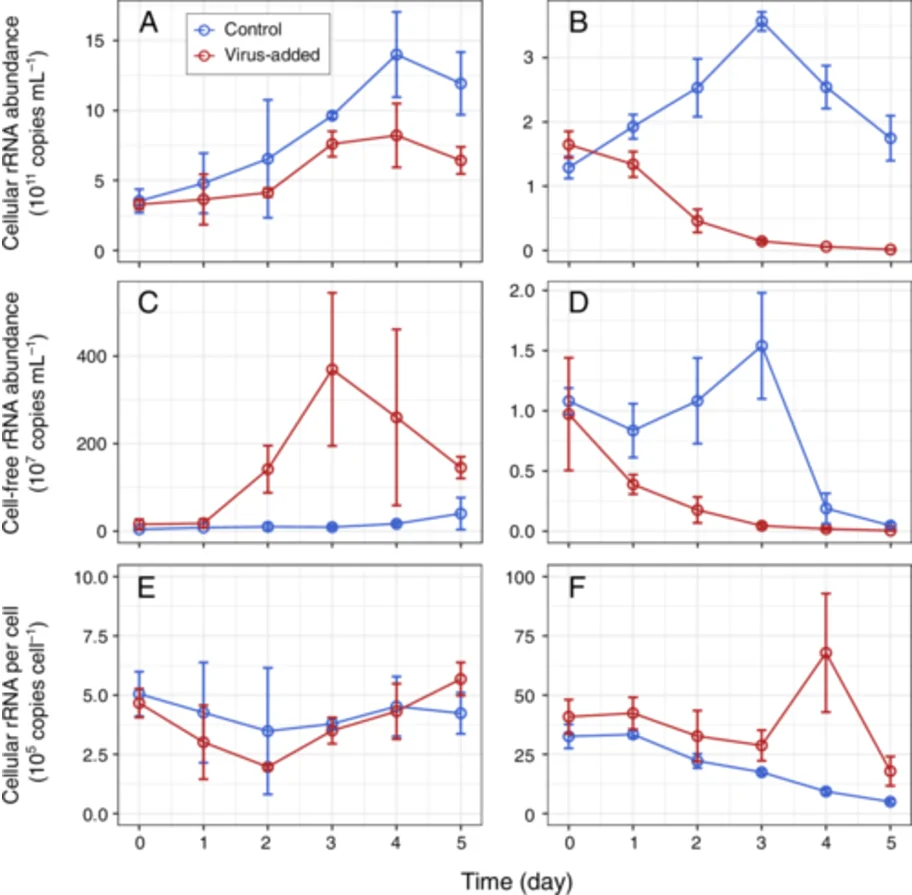
Temporal changes in cellular (A, B) and cell‐free 18S rRNA (C, D), and per‐cell rRNA content (E, F). Left and right panels denote the data from *Chaetoceros tenuissimus* and *Heterosigma akashiwo* experiments, respectively. Error bars denote ±1 SD (*n* = 3). rRNA, ribosomal RNA.

In *C. tenuissimus*, the initial abundance of cellular rRNA was 3.53 ± 0.84 × 10^11^ copies mL^–1^ (mean ± SD). In the control treatment, the value increased until day 4, when it reached a maximum of 1.40 ± 0.30 × 10^12^ copies mL^–1^ (Figure 3A). In the virus‐added treatment, the cellular rRNA increased until day 4, but the values were significantly lower than those of the control after day 3 (Holm‐adjusted *p* < 0.05). The concentrations of cell‐free rRNA were kept at low values (< 4.04 ± 3.65 × 10^8^ copies mL^–1^) in the control treatment, while the values increased sharply in the virus‐added treatment until reaching maxima at day 4 (3.70 ± 1.75 × 10^9^ copies mL^–1^) (Holm‐adjusted *p* < 0.05) (Figure 3C). The mean number of 18S rRNA per *C. tenuissimus* cell ranged between 1.97 × 10^5^ and 5.68 × 10^5^ copies cell^–1^ (Figure 3E). Although there was a 2.8‐fold difference between the maximum and minimum values in each time and condition, no significant effect of time and treatment was detected by two‐way repeated measures ANOVA (*p* > 0.05).

In *H. akashiwo*, the initial abundance of cellular rRNA was 1.29 ± 0.17 × 10^11^ copies mL^–1^. In the control treatment, the value increased until day 3 (3.57 ± 0.15 × 10^11^ copies mL^–1^) and then decreased to the end of the experiment (Figure 3B). In the virus‐added treatment, the cell‐free rRNA concentration decreased consistently from the start to the end of the experiment and showed lower values than the control (Holm‐adjusted *p* < 0.05) (Figure 3D). The mean number of 18S rRNA per *H. akashiwo* cell varied between 0.50 × 10^6^ and 6.78 × 10^6^ copies cell^–1^ (Figure 3F). The per‐cell concentrations of 18S rRNA were 1.3–1.7 times higher in the virus‐added treatments than in the control until day 3, and 3.6–7.3 times higher at later time periods (Holm‐adjusted *p* < 0.05).

**Figure 4:**
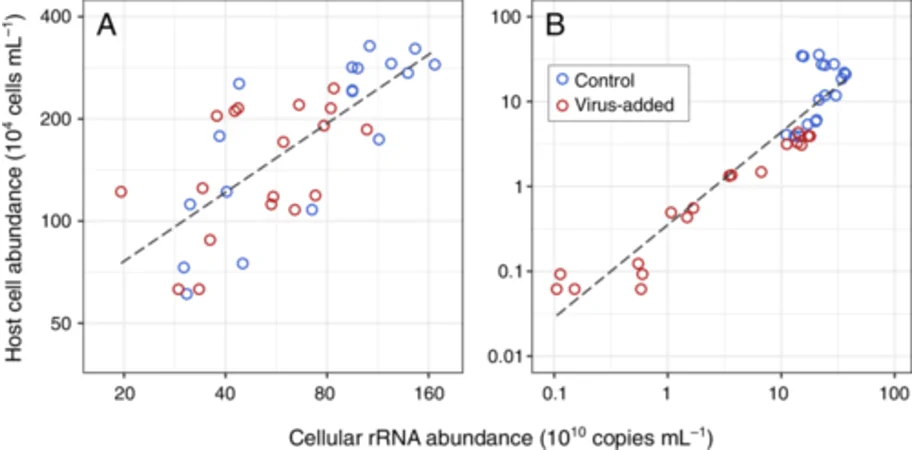
Relationship between algal cell concentration and the cellular rRNA concentration in the medium. The left (A) and right (B) panels denote the data from *Chaetoceros tenuissimus* and *Heterosigma akashiwo* experiments, respectively. rRNA, ribosomal RNA.

Despite the variation of per‐cell rRNA content, significant positive correlations were observed between cellular rRNA concentration and cell concentration in both species, with the correlation coefficients (*r*) of 0.72 and 0.94 for *C. tenuissimus* and *H. akashiwo*, respectively (Pearson's test, *p* < 0.001, *n* = 36) (Figure 4A,B).

### 3.3 Viral Proliferation and Temporal Changes in Viral Marker Genes

The concentrations of infectious virus particles increased from 5.8 × 10^5^ to 1.9 × 10^8^ MPN mL^−1^ for CtenRNAV‐II and from 7.6 × 10^5^ to 1.4 × 10^7^ MPN mL^−1^ for HaV120 between days 0 and 5. These increases were consistent with the production of infectious progeny viruses in both host–virus systems.

**Figure 5:**
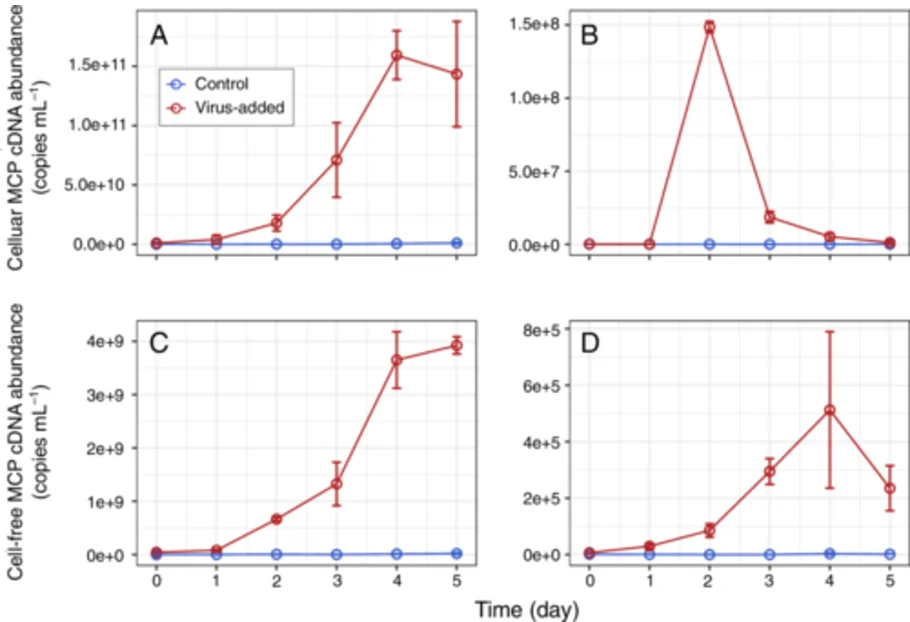
Temporal changes in viral MCP cDNA abundances in cellular (A, B) and cell‐free (C, D) fractions. Left and right panels denote the data from *Chaetoceros tenuissimus* and *Heterosigma akashiwo* experiments, respectively. Error bars denote ±1 SD (*n* = 3). cDNA, complementary DNA; MCP, major capsid protein.

In the *C. tenuissimus* experiment, the MCP cDNA abundance increased after day 1 and reached a maximum on day 4 in the cellular fraction (Figure 5A). In the cell‐free fraction, the abundance of these cDNAs sharply increased after day 2 and reached a maximum on day 5 (Figure 5C). In the *H. akashiwo* experiment, the MCP cDNA abundance peaked at day 2 in the cellular fraction, while that in the cell‐free fraction peaked at day 4 (Figure 5B,D).

### 3.4 Degradation of Cell‐Free rRNA

**Figure 6:**
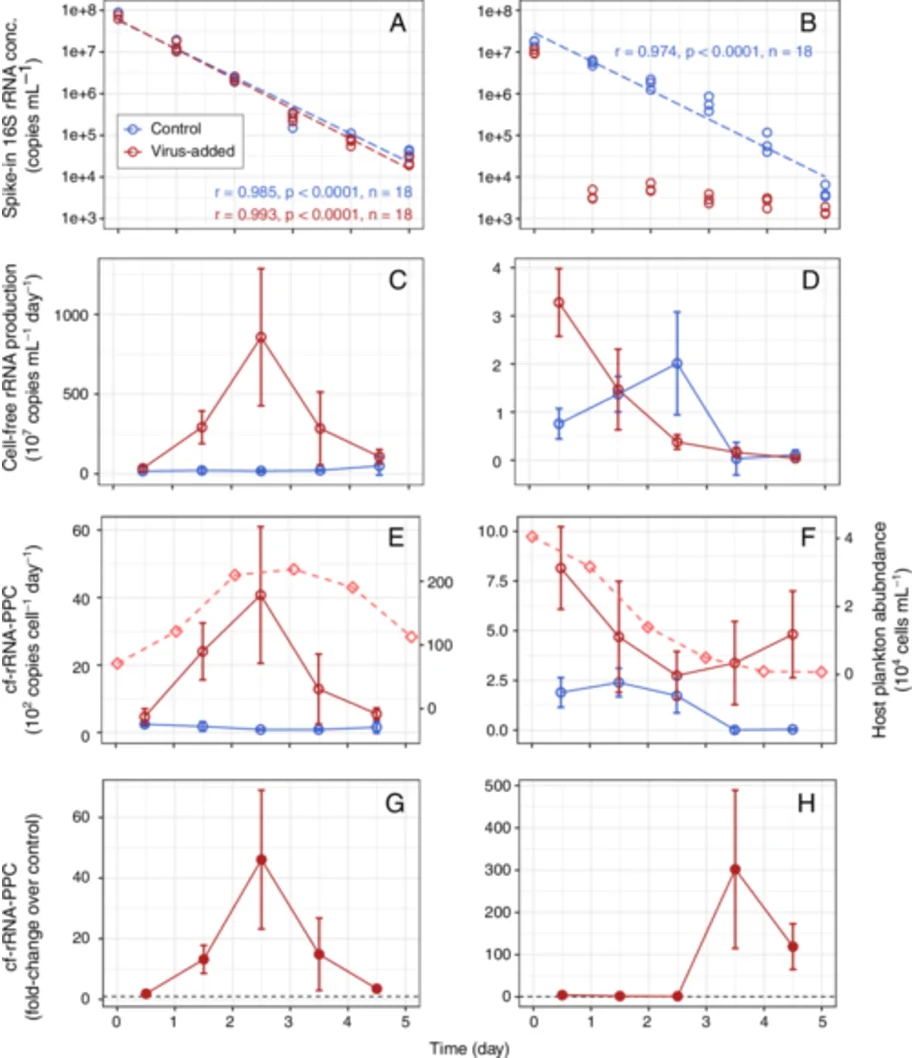
Temporal changes in spike‐in 16S rRNA abundances in the cell‐free fraction (A, B) and measures of host cell lysis. Measures of cell lysis are shown as cell‐free 18S rRNA production (C, D) and cell‐free rRNA production per cell (cf‐rRNA‐PPC) (E, F). Fold‐changes of cf‐rRNA‐PPC in virus‐added treatments over control treatment (control = 1) (G, H). Dashed lines in figures (E) and (F) indicate the mean host cell abundance in the virus‐added treatment. Left and right panels denote the data from *Chaetoceros tenuissimus* and *Heterosigma akashiwo* experiments, respectively. Error bars denote ±1 SD (*n* = 3). rRNA, ribosomal RNA.

In the *C. tenuissimus* experiment, the concentration of spike‐in 16S rRNA in the medium decreased exponentially with time (i.e., the degradation rate was linearly proportional to rRNA concentration) in both control and virus‐added treatments (Pearson's test, *p* < 0.01, *n* = 18) (Figure 6A). The decay constant was around 1.78–2.16 day^–1^ (corresponds to half‐lives of 7.7–9.3 h) until day 3, then decreased to 0.83–1.37 day^–1^ (half‐lives of 12.1–20.0 h).

In the *H. akashiwo* experiment, spike‐in 16S rRNA decreased similarly to that in the *C. tenuissimus* experiment in the control treatment (Pearson's test, *p* < 0.01, *n* = 18), with the decay constant around 1.09–2.67 day^–1^ (half‐lives of 6.2–15.3 h) (Figure 6B). On the other hand, in the virus‐added treatment, spike‐in 16S rRNA decreased steeply after virus addition and reached the lower limit of the quantification on day 1. For this treatment, we used a decay constant of 8.43 day^–1^ (half‐life of 2.0 h), calculated from the concentration difference between days 0 and 1, for the entire sampling period.

### 3.5 Cell‐Free rRNA Production

In the *C. tenuissimus* experiment, the production rate of cell‐free rRNA (release from the cellular fraction) increased and reached a maximum between days 2 and 3 in the virus‐added treatment and decreased at later time periods, while it was consistently low in the control treatment (Figure 6C). The values were significantly higher in the virus‐added treatment than the control during days 1–3 (Holm‐adjusted *p* < 0.05). Between days 2 and 3, the cf‐rRNA‐PPC was 46.1 ± 22.9‐fold higher (mean ± SD) in the virus‐added treatment than in the control (Figure 6G).

In the *H. akashiwo* experiment, cell‐free rRNA production in the virus‐added treatment was significantly higher than that in the control between days 0 and 1 (Holm‐adjusted *p* < 0.05), while the values were not significant between days 2–5 due to the significant difference in cell density (Figure 6D). On the other hand, the cf‐rRNA‐PPC was significantly higher in the virus‐added treatment between days 0–1 and 4–5 (Holm‐adjusted *p* < 0.05) (Figure 6F), with the values 302.1 ± 187.3‐fold higher than in the control treatment between days 3 and 4 (Figure 6H).

## 4 Discussion

### 4.1 Cell‐Free rRNA as a Proxy of Plankton Cell Lysis

Our experiments provide direct evidence that cell‐free rRNA can serve as a quantitative indicator of viral lysis in eukaryotic microalgae. In both *C. tenuissimus* and *H. akashiwo*, viral infection induced marked increases in cell‐free 18S rRNA release per cell, up to 46.1‐ and 302.1‐folds, respectively, relative to the noninfected controls (Figure 6E–H). In control treatments, cell‐free rRNA production remained low and relatively stable despite active host growth, indicating that intact cells do not release substantial amounts of rRNA into the surrounding medium. Our results extend previous observations in prokaryotes (Zhong et al. 2023) to eukaryotic phytoplankton and provide a key methodological foundation for applying MoRS to microeukaryotic plankton communities.

For both plankton species, significant positive correlations were observed between the rRNA concentration in cell fractions and cell density, indicating that rRNA abundance serves as an indicator of plankton biomass at the population scale (Figure 4). On the other hand, the number of ribosomes per cell varied during the incubation up to 3–7‐fold within the same species (Figure 3E,F). The variations in ribosome content were small relative to those in production rate and would not significantly affect the conclusions; however, caution is required when using rRNA as an indicator of lysis. For example, it has been reported that infected marine bacterial cells actively reduce their ribosome content to extremely low levels, possibly to prevent the synthesis of phage proteins (Brüwer et al. 2024). In such cases, rRNA may decrease its function as a lysis marker.

Another key finding from our experiments was the importance of monitoring the degradation of cell‐free rRNA. The spike‐in rRNA exhibited exponential decay in the medium, indicating that cell‐free rRNA degradation follows first‐order kinetics (Figure 6A,B). Similar first‐order degradation has also been reported for environmental DNA (Mauvisseau et al. 2022). Since the rate equation we derived assumes first‐order kinetics of cell‐free rRNA degradation, this result supports the validity of our approach for estimating cell‐free rRNA production. A previous study demonstrated that the decay constant varies with environmental factors, including temperature, light, and bacterial abundance (Endo et al. 2025). Therefore, when applying this approach to natural plankton communities, it is important to measure the cell‐free rRNA degradation constant for each seawater sample. Moreover, because the amount of cell‐free rRNA is influenced by both the host cell density and the cellular rRNA content of individual cells, caution is required when comparing cell‐free rRNA production among species. Normalizing cell‐free rRNA production by host cell abundance (or another proxy for host abundance) is therefore recommended to minimize these effects. Another possible limitation of this approach is that the degradation kinetics of *E. coli* 16S rRNA may not exactly match those of eukaryotic 18S rRNA. Further studies should evaluate the degradation kinetics using purified ribosomes from representative plankton.

In our study, virus‐added treatment of *H. akashiwo* showed a much higher decay constant, likely because a large number of bacteria were introduced along with the virus lysate, as evidenced by flow cytometry (Figures 2G and 6B). As a result, the apparent concentration of cell‐free rRNA was lower in the virus‐added treatment than in the control; however, cf‐rRNA‐PPC, accounting for the decay constant and cell density, consistently showed higher values in the virus‐added treatment (Figure 6F). In this experiment, as the spike‐in rRNA in the virus‐added treatment was already depleted by day 1, interval‐specific degradation constants could not be reliably estimated. We therefore applied the degradation constant estimated during the first 24 h throughout the incubation, treating it as a conservative 24‐h‐averaged estimate. If the spike‐in rRNA was depleted well before the first 24‐h sampling point, or if degradation accelerated following bacterial proliferation (Figure 2G), the actual degradation constant might have been larger than estimated here, resulting in higher cell‐free rRNA production rates.

### 4.2 Differential Infection Dynamics of Two Algal Host–Virus Systems

Despite the general applicability of cell‐free rRNA as a lysis indicator, the temporal dynamics of rRNA release differed markedly between the two host–virus systems examined, highlighting distinct infection strategies. In the *C. tenuissimus* and CtenRNAV system, viral infection did not affect host growth until day 3, and the proxies of plankton biomass (cell count, chlorophyll *a*, and cellular rRNA) increased until day 3 or 4 (Figures 2A,C, and 3A), while the peak of cell lysis occurred between days 2 and 3 (Figure 6C,E). Viral marker genes in the cellular fraction also accumulated progressively over days 1–4 (Figure 5A), indicating active viral replication preceding large‐scale lysis. Also, viral MCP cDNA accumulated in the cell‐free fraction during the experiment, indicating that viral particles and transcripts were released into the medium (Figure 5C). The temporal decoupling between cell lysis and population decline suggests that active viral lysis can occur while host populations are still growing. This finding has important implications for interpreting field observations, as a single population can contribute to DOM production via cell lysis even during the apparent growth phase.

In contrast, the *H. akashiwo* and HaV system exhibited a fundamentally different trajectory. The concentrations of cells, chlorophyll *a*, and cellular rRNA declined immediately after viral addition (Figures 2B,D and 3B), and most *H. akashiwo* cells lost motility within 24 h of virus inoculation as previously reported (Nagasaki et al. 1999). These results indicate that host cells experienced rapid physiological changes and growth suppression. Regarding the cell lysis, elevated per‐cell‐free rRNA production was observed during both the early (days 0 and 1) and late (days 3–5) phases of infection (Figure 6F). The HaV MCP gene was expressed intensively in the host cell on day 2, followed by accumulation in the dissolved fraction around day 4 (Figure 5B,D), indicating that host cell lysis by viral maturation occurred in the late phase. However, increased cell lysis in the early stage cannot be explained by canonical viral lysis but rather by loss of cellular integrity prior to viral replication.

Such early‐stage mortality may arise from multiple processes, including abortive infection, membrane destabilization during viral entry, or bacterial‐induced cell damage. In bacteriophage systems, a related phenomenon termed “lysis from without” has been documented, in which excessive adsorption of viral particles leads to rapid cell disruption by lysozyme activity without productive replication (Abedon 2011; Delbrück 1940). While this phenomenon is not formally defined in microeukaryotic virus systems, similar nonproductive mortalities at high viral concentrations have been observed in dsDNA viruses infecting marine protists, such as *Aureococcus anophagefferens* (Arrigo et al. 2014; Moniruzzaman et al. 2018). Therefore, multiple viral attachments in the early phase may damage host cells in our experiment, as a relatively high MOI (10) was used to synchronize infection across the host population. Another possibility is that bacteria introduced together with the nonaxenic viral lysate contributed to early host mortality. Numerous marine bacteria impair algal physiology and promote algal cell death through both direct and indirect interactions (Barak‐Gavish et al. 2018; Wang et al. 2026). Thus, a fraction of host cells may undergo premature death, releasing intracellular components, such as rRNA, into the cell‐free fraction without maturation of progeny viruses.

We quantified viral MCP cDNA abundance as an indicator of active viral replication and release into the medium. However, the temporal dynamics of cell‐free rRNA release could not be precisely aligned with progeny virion production because viral particle abundance was not monitored at intermediate time points during the incubation. Future studies incorporating time‐resolved viral particle counts or infectivity assays will further strengthen the mechanistic interpretation of cell‐free rRNA production.

### 4.3 Implications for Marine Ecology

Our study has the potential to provide a taxon‐resolved, quantitative measure for lytic cell mortality, addressing a long‐standing challenge in plankton ecology. For estimating plankton mortality, the dilution assay was frequently used (Kimmance and Brussaard 2010). Although this method offers a significant advantage for assessing both grazing and viral lysis simultaneously, it is often labor‐intensive and lacks detailed taxonomic resolution. The method developed in this study can serve as a useful complement to existing techniques due to its ease of use and taxonomic comprehensiveness. Cell‐free rRNA quantification, when combined with field incubation and high‐throughput sequencing, enables simultaneous assessment of lysis across diverse taxa in natural communities.

On the other hand, further investigation is required to determine how the magnitude of observed cell lysis influences biogeochemical cycling. In addition to ribosomes quantified in this study, cell lysis releases a wide range of intracellular biomolecules into the surrounding environment. It has been estimated that viral lysis can contribute up to ~45% of the labile DOC pool in marine systems (Moran et al. 2022). These released compounds are rapidly utilized by heterotrophic bacteria and can subsequently be transformed into more recalcitrant forms of DOM through microbial processing (Jiao et al. 2010, 2024). However, the relationship between the taxonomic composition of lysed organisms and the chemical composition of released DOC remains poorly understood, largely due to analytical limitations in resolving molecular‐level complexity.

Furthermore, in natural environments, observed cell lysis is not necessarily attributable solely to viral infection. Sloppy feeding, parasitic interactions, and algicidal bacteria can also induce cell damage and lead to the release of intracellular rRNA and other cellular components (Chambouvet et al. 2008; Coyne et al. 2022; Saba et al. 2011). To achieve a mechanistic understanding of cell lysis processes in marine ecosystems, it is essential to adopt integrative approaches that combine molecular analyses, such as gene expression profiling, with direct observations using microscopy.

## Author Contributions

**Saki Kikuya:** conceptualization, writing – original draft, writing – review and editing, methodology, investigation, data curation, formal analysis, visualization. **Yuji Tomaru:** supervision, methodology, resources, writing – review and editing. **Keizo Nagasaki:** methodology, supervision, resources, writing – review and editing. **Daichi Morimoto:** methodology, writing – review and editing. **Yuki Yamagishi:** methodology, writing – review and editing, investigation. **Hiroyuki Ogata:** supervision, funding acquisition, writing – review and editing. **Hisashi Endo:** conceptualization, methodology, data curation, formal analysis, writing – original draft, funding acquisition, visualization, supervision, validation, investigation.

## Ethics Statement

The authors have nothing to report.

## Conflicts of Interest

None declared.

## Supporting information


Supporting File 1

Supporting File 2

Supporting File 3

## Data Availability

Environmental DNA (EDN3). The metadata describing the experimental design, sampling, and the collected variables, along with their values, are available as supplementary data.
